# Laryngeal non-Hodgkin lymphoma: Report of four cases and review of the literature

**DOI:** 10.1515/biol-2022-0937

**Published:** 2024-09-09

**Authors:** Xin Tang, Dingting Wang, Huajun Feng, Gang Qin

**Affiliations:** Department of Otolaryngology Head and Neck Surgery, The Affiliated Hospital of Southwest Medical University, No. 25 Taiping Street, Jiangyang District, Luzhou, Sichuan, 646000, China; Department of Otolaryngology Head and Neck Surgery, The Affiliated Hospital of Southwest Medical University, Luzhou, Sichuan, 646000, China

**Keywords:** larynx, MALT lymphoma, radiotherapy, chemotherapy, surgery

## Abstract

Non-Hodgkin lymphoma (NHL) limited to the larynx is very rare. The authors present the clinical diagnosis and treatment of four patients with laryngeal NHL. Case 1 was diagnosed with glottic, subglottic, and tracheal mucosa-associated lymphoid tissue (MALT) lymphoma, and was treated with radiotherapy and chemotherapy after surgery. Case 2 was diagnosed with laryngeal MALT lymphoma and underwent radiotherapy. Case 3 was diagnosed with angioimmunoblastic T-cell lymphoma, and was treated with radiotherapy and chemotherapy. Case 4 had MALT lymphoma in the glottic area with a malignant thyroid tumor, and was treated with radiotherapy and chemotherapy after surgery. More reports and research on this disease are needed.

## 1 Introduction

Mucosa-associated lymphoid tissue (MALT) lymphoma is B-cell non-Hodgkin lymphoma (NHL) that is mainly located in the stomach. Since Diebold et al. [1] described MALT lymphoma as a separate lymphoma in 1990, only about 90 cases had been reported in the English-language literature through 2020 [2]. Angioimmunoblastic T-cell lymphoma (AITL) is an invasive T-cell lymphoma. Since AITL was included in the revised classification of lymphoid neoplasms in Europe and the United States in 1994, few cases of laryngeal AITL have been reported. This article describes the clinical diagnosis and treatment of three patients with laryngeal MALT lymphoma and one patient with AITL, and the relevant literature is reviewed to provide a reference for clinical diagnosis and treatment.

## 2 Case report

### 2.1 Case 1

**Figure 1:**
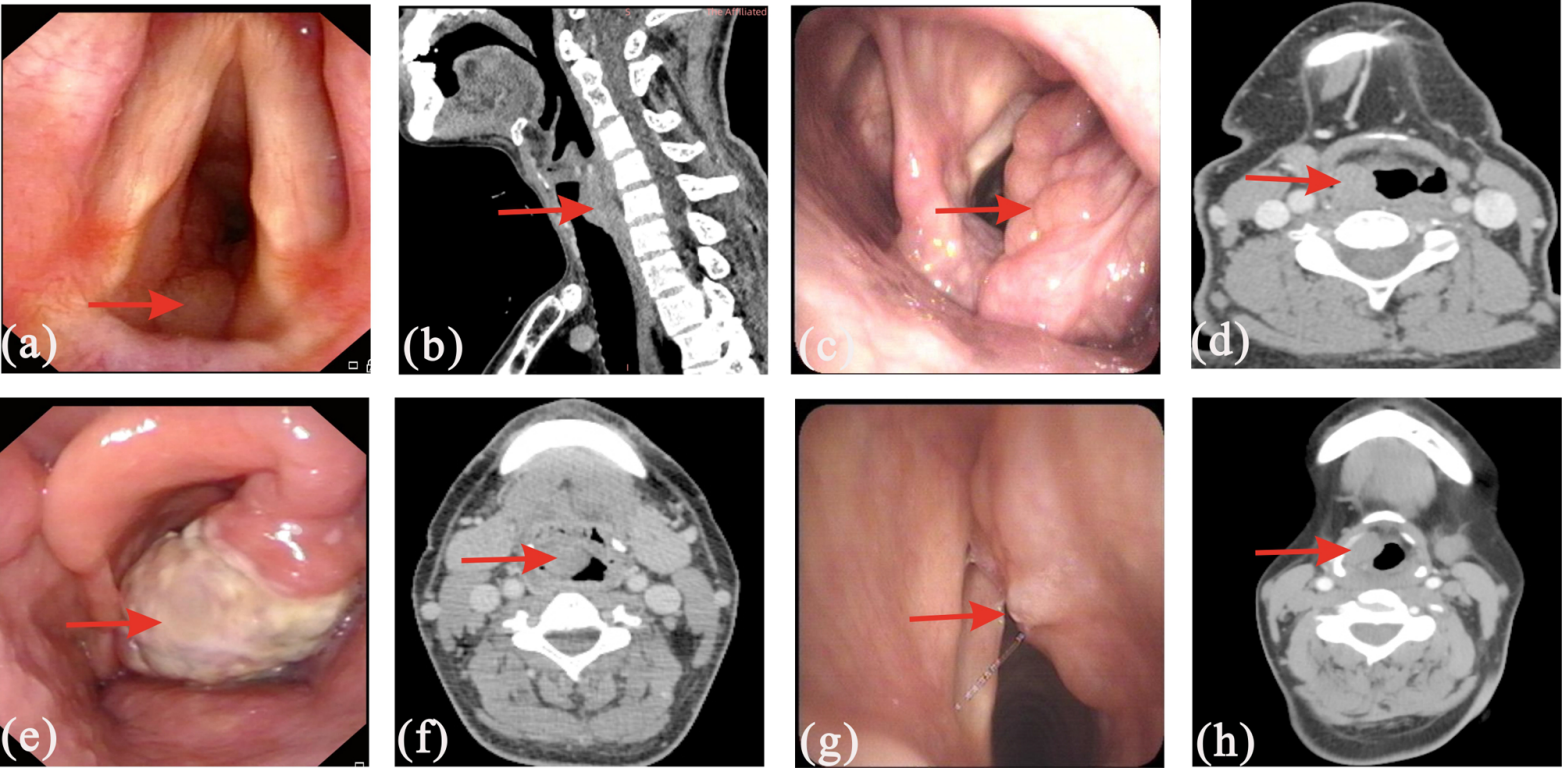
(a) Laryngoscopy indicated a non-smooth neoplasm in the subglottic region. (b) CT image indicating a mass at the glottis and subglottis and opening of the trachea. (c) Laryngoscopy indicated a mass in the right aryepiglottic fold. (d) CT image indicating a mass in the right supraglottic portion. (e) Laryngoscopy indicated a non-smooth neoplasm in the right aryepiglottic fold. (f) CT image indicating a mass in the right glottic portion. (g) Laryngoscope indicated a non-smooth neoplasm in the right ventricular bands. (h) CT image indicating a mass in the right supraglottic portion.

A 55-year-old man presented with cough, shortness of breath, and difficulty breathing for 9 months, with worsening symptoms in the previous 2 months. He denied any tobacco or alcohol use, and his family history was unremarkable. Laryngoscopy revealed a light-red and non-smooth neoplasm in the subglottic region (Figure 1a). Contrast-enhanced computed tomography (CT) of the neck revealed a soft tissue mass with a clear boundary and heterogeneous thickening at the glottis and subglottis and opening of the trachea, and no enlarged lymph nodes were found in the neck (Figure 1b). Enhanced magnetic resonance imaging (MRI) of the neck revealed a heterogeneous thickening, clear boundary, and significantly uniformly enhanced soft tissue at the glottis, subglottis, and opening of the trachea, with an iso T1 signal and a slightly longer T2 signal. Positron emission tomography-computed tomography (PET-CT) revealed a heterogeneous thickened soft tissue with slightly increased glucose metabolism at the glottis and subglottis and opening of the trachea, and no tumor lesions with increased glucose metabolism were found in other locations (PET-CT results for this patient have been reported [3]).

The diagnosis was not confirmed by bronchofibrescopic biopsy, so the patient underwent tracheotomy and laryngeal dehiscence surgery to resect the laryngeal and tracheal tumors. Postoperative immunohistochemistry (IHC) results of the laryngeal tumor were as follows: cluster of differentiation 20 (CD20) (partial +), CD79α (diffuse +), CD5 (−), CD10 (−), CyclinD1 (−), B-cell lymphoma 2 (Bcl-2) (+), Bcl-6 (−), and Epstein Barr encoding region (EBER) *in situ* hybridization (−). The gene rearrangement results were as follows: clonal amplification peaks were observed for IgH and IgK gene rearrangement; no clonal amplification peak was observed for IgL gene rearrangement. The diagnosis of MALT lymphoma was confirmed. Local palliative intensity-modulated radiation therapy (IMRT) (30–36 Gy) was performed after surgery. The tracheal tube was successfully removed 5 months after surgery, and no recurrence was observed in 30 months of follow-up.

### 2.2 Case 2

A 58-year-old woman presented with hoarseness for 1 month. She denied any tobacco or alcohol use, and her father died of a rectal tumor. Laryngoscopy revealed a smooth and irregular neoplasm in the right aryepiglottic fold and ventricular bands (Figure 1c). Contrast-enhanced CT of the neck revealed a soft tissue mass with homogeneous enhancement at the bilateral vocal cords, ventricular bands, and right aryepiglottic fold, and multiple enlarged lymph nodes were seen in the bilateral submaxillary and carotid sheath areas (Figure 1d). Enhanced MRI of the neck revealed an irregular thickening of soft tissue at the lateral and posterior walls of the laryngopharynx, with an iso T1 signal and a high T2 signal. Multiple enlarged lymph nodes were seen in the bilateral submaxillary and carotid sheath areas. PET-CT revealed a soft tissue with increased glucose metabolism in the vocal cords, ventricular band, posterior wall of the laryngopharynx, and right epiglottis, and no tumor lesions with increased glucose metabolism were found in other locations.

After biopsy of laryngeal under general anesthesia, the IHC results were as follows: CD20 (+), CD79α (+), CD5 (+), CD10 (−), CD23 (partial +), CyclinD1 (−), Bcl-2 (+, 60%), Bcl-6 (−), and EBER ISH (−). The gene rearrangement results were as follows: clonal amplification peaks were observed for IgH and IgK gene rearrangement; no clonal amplification peak was observed for IgL gene rearrangement. The diagnosis of MALT lymphoma was confirmed. The clinical stage was evaluated as stage IE, according to the Ann Arbor staging system. The treatment plan was local IMRT (36–45 Gy/20 F/4 W). No recurrence was observed in 17 months of follow-up.

### 2.3 Case 3

A 54-year-old man presented with repeated sore throat for 1 year and swallow obstruction for 20 days. He denied any tobacco or alcohol use, and his family history was unremarkable. Laryngoscopy revealed a neoplasm in the right aryepiglottic fold, pyriform sinus, and epiglottis (Figure 1e). Contrast-enhanced CT of the neck revealed an irregular and homogeneous enhanced soft tissue mass in the laryngopharynx (from the C3 to C6 vertebrae), and multiple lymph nodes were seen in the bilateral jugular vein chain (Figure 1f). Enhanced MRI of the neck revealed an irregular thickening, unclear boundary, and significantly homogeneous enhanced soft tissue at the epiglottis to the glottic portion, with an iso T1 signal and a longer T2 signal. Enhanced lymph nodes were seen in the right carotid sheath and posterior cervical triangle. PET-CT revealed increased glucose metabolism in the laryngeal soft tissue mass and bilateral jugular chain lymph nodes, but no tumor lesion with increased glucose metabolism was found in other locations.

After biopsy of laryngeal under general anesthesia after tracheotomy, the IHC results were as follows: Bcl-6 (partial +), CD10 (+), PD-1 (+), CD30 (−), CD3ε (+), CD7 (+), CD43 (+), CD45RO (+), CD56 (−), Granzyme B (−), TIA-1 (partial +), Ki-67 (+), and EBER ISH (−). The diagnosis of AITL was confirmed. The clinical stage was evaluated as stage II, according to the Ann Arbor staging system. The patient was treated with a combination chemotherapy regimen (CHOEP) in six sessions and local radiotherapy (50–60 Gy/28 F). Relapse of the lymphoma was the cause of his death, 18 months after the initial diagnosis.

### 2.4 Case 4

A 45-year-old woman presented with hoarseness for 10 months and a mass in her neck for 20 days. She denied any tobacco or alcohol use, and her family history was unremarkable. Laryngoscopy revealed a non-smooth neoplasm in the right paraglottic space (Figure 1g). Contrast-enhanced CT of the neck revealed a limited enhanced soft tissue mass at the right vocal cords and aryepiglottic fold, and slightly hypodense nodules was seen in the right lobe of the thyroid gland (Figure 1h). Enhanced MRI of the neck revealed a heterogeneously enhanced soft tissue at the right sidewall of the laryngeal vestibule, with a longer T1 signal and a slightly longer T2 signal. Thyroid ultrasound revealed a thyroid imaging reporting and data system (T1-RADS) 4B nonuniform echo nodule in the right lobe of the thyroid gland and a T1-RADS 4A hypoechoic nodule in the left lobe. And cytology puncture was considered for papillary carcinoma.

She underwent total thyroidectomy and laryngeal dehiscence surgery to resect the laryngeal tumor at another hospital. Postoperative IHC results of the laryngeal tumor were as follows: CD20 (+), CD79a (+), CD3 (−), CD10 (−), Bcl-2 (+), CD5 (−), Cyclin D1 (−), and CD23 (−). She was diagnosed with thyroid malignancy and laryngeal MALT lymphoma. The patient was treated with a combination chemotherapy regimen (CHOP) and local radiotherapy (24–30 Gy). The tracheal tube was successfully removed 11 months after surgery, and no recurrence was observed in 36 months of follow-up.


**Informed consent:** Informed consent has been obtained from all individuals included in this study.
**Ethical approval:** The research related to human use has been complied with all the relevant national regulations, institutional policies and in accordance with the tenets of the Helsinki Declaration, and has been approved by the authors’ institutional review board or equivalent committee.

## 3 Discussion

Laryngeal lymphoma is mainly NHL, the most common subtype is B-cell NHL, and the incidence of T-cell type and natural killer cell NHL is about 10% [4]. AITL is one of the most common types of T-cell lymphoma and is very rare in the larynx. AITL is an aggressive lymphoma whose clinical manifestations are mainly progressive lymph node enlargement, hepatosplenomegaly, and systemic symptoms, and 90% of patients have advanced disease at diagnosis [5]. The main symptoms of primary NHL in the larynx are local symptoms, such as hoarseness, dysphagia, and dyspnea. Both the clinical symptoms of the AITL diagnosed here and the laryngeal AITL reported by Konstantinos were mainly local symptoms, and they both had a lower stage II stage [6]. Laryngeal NHL is mostly located in the supraglottic region, possibly because of the richness of lymphoid tissue in the supraglottic area, followed by the glottic region and the subglottic region. Under endoscopy, laryngeal NHL is mostly a smooth nonulcerative mass, and imaging manifestations are similar to those of malignant tumors without specificity. However, these images can be used to assess the extent of tumor invasion. The diagnostic value of PET-CT for laryngeal NHL remains controversial [7,8]. Of the four patients with laryngeal NHL in this study, three had laryngeal MALT lymphoma, and one had AITL. The lesions were mostly located in the supraglottic region. In Case 1, the lesion was located in the glottic and subglottic regions and involved the trachea.

Primary NHL of the larynx is rare, and there is no standard treatment. The currently reported treatment options for NHL confined to the larynx include laser or laryngeal split surgery alone, chemoradiotherapy alone, and surgery combined with chemoradiotherapy alone, and most patients have a good prognosis [9–11]. Surgery is controversial. Kania et al. proposed that for completely surgically resectable laryngeal NHL, surgical treatment is feasible [12]. For subglottic stenosis caused by subglottic NHL, Alexander et al. reported laryngeal dehiscence surgery for the total resection of laryngeal tumors [13–15]. Gonzalez-Murillo et al. reported that laryngeal NHL was treated with surgery and chemoradiotherapy [16]. Some scholars also propose that for patients with acute laryngeal obstruction or massive hemorrhage, tumor reduction surgery can be performed after the establishment of a safe airway [6]. Of the four cases reported in this article, Case 4 received surgical treatment at another hospital. Case 1 had subglottic stenosis caused by subglottic MALT lymphoma. Laryngoscopy and imaging findings suggested that the tumor was limited and had a clear boundary. Therefore, complete tumor resection was possible. Therefore, in this article, laryngeal dehiscence surgery was chosen to resect laryngeal tumors after tracheotomy to establish a safe airway. Preoperative examinations of the other two patients showed that the range of involvement was extensive. So only biopsy was performed.

Radiation therapy and chemotherapy are the most common therapeutic strategies advocated for the treatment of the primary laryngeal lymphomas. For laryngeal MALT lymphoma, a moderate dose of locoregional radiotherapy has been reported to achieve complete remission [17]. And the efficacy of chemotherapy remains controversial [18,19]. AITL has a low incidence and no accepted standard treatment regimen. The recurrence rate is high, and the long-term efficacy of AITL is poor. The first-line chemotherapy regimen currently recommended by AITL is based on either CHOP or CHOPE, and chemotherapy options incorporating other biological agents are still under investigation [20]. For laryngeal AITL, previous reports by Markou et al. [6] and the laryngeal AITL patients reported in this article were treated with a combination chemotherapy regimen (CHOP) and radiotherapy. The prognosis of laryngeal NHL patients is closely related to the pathological classification of NHL, and the survival rate of patients with B-cell NHL is better than that of patients with T-cell NHL [4,21]. Of the four patients in this study, the patient who was diagnosed with laryngeal AITL died of tumor recurrence, and the remaining patients were alive.

## 4 Conclusion

The clinical symptoms, laryngoscopy, and imaging findings of laryngeal NHL are nonspecific, and clinicians should be vigilant about the diagnosis of laryngeal NHL. More case reports and studies are needed on the diagnosis and treatment of this rare disease.
